# Digital Health Apps in the Clinical Care of Inflammatory Bowel Disease: Scoping Review

**DOI:** 10.2196/14630

**Published:** 2019-08-19

**Authors:** Andrew Lukas Yin, David Hachuel, John P Pollak, Ellen J Scherl, Deborah Estrin

**Affiliations:** 1 Medical College Weill Cornell Medicine New York, NY United States; 2 Cornell Tech New York, NY United States; 3 augGI Technologies New York, NY United States; 4 Jill Roberts Center for Inflammatory Bowel Disease Weill Cornell Medicine New York, NY United States

**Keywords:** digital health, mHealth, mobile health, mobile technology, smartphone, eHealth, review, inflammatory bowel disease, Crohn’s disease, ulcerative colitis

## Abstract

**Background:**

Digital health is poised to transform health care and redefine personalized health. As Internet and mobile phone usage increases, as technology develops new ways to collect data, and as clinical guidelines change, all areas of medicine face new challenges and opportunities. Inflammatory bowel disease (IBD) is one of many chronic diseases that may benefit from these advances in digital health. This review intends to lay a foundation for clinicians and technologists to understand future directions and opportunities together.

**Objective:**

This review covers mobile health apps that have been used in IBD, how they have fit into a clinical care framework, and the challenges that clinicians and technologists face in approaching future opportunities.

**Methods:**

We searched PubMed, Scopus, and ClinicalTrials.gov to identify mobile apps that have been studied and were published in the literature from January 1, 2010, to April 19, 2019. The search terms were (“mobile health” OR “eHealth” OR “digital health” OR “smart phone” OR “mobile app” OR “mobile applications” OR “mHealth” OR “smartphones”) AND (“IBD” OR “Inflammatory bowel disease” OR “Crohn's Disease” (CD) OR “Ulcerative Colitis” (UC) OR “UC” OR “CD”), followed by further analysis of citations from the results. We searched the Apple iTunes app store to identify a limited selection of commercial apps to include for discussion.

**Results:**

A total of 68 articles met the inclusion criteria. A total of 11 digital health apps were identified in the literature and 4 commercial apps were selected to be described in this review. While most apps have some educational component, the majority of apps focus on eliciting patient-reported outcomes related to disease activity, and a few are for treatment management. Significant benefits have been seen in trials relating to education, quality of life, quality of care, treatment adherence, and medication management. No studies have reported a negative impact on any of the above. There are mixed results in terms of effects on office visits and follow-up.

**Conclusions:**

While studies have shown that digital health can fit into, complement, and improve the standard clinical care of patients with IBD, there is a need for further validation and improvement, from both a clinical and patient perspective. Exploring new research methods, like microrandomized trials, may allow for more implementation of technology and rapid advancement of knowledge. New technologies that can objectively and seamlessly capture remote data, as well as complement the clinical shift from symptom-based to inflammation-based care, will help the clinical and health technology communities to understand the full potential of digital health in the care of IBD and other chronic illnesses.

## Introduction

Digital health technologies—tools leveraging mobile phones, tablets, Web platforms, and wearables to improve health outcomes—are rapidly changing the practice of medicine and redefining approaches to health care. By the end of 2018, 67% of the global population (5.1 billion people) subscribed to mobile Internet services, a number expected to increase to 71% (5.8 billion people) by 2025 [1]. In 2013, the Pew Research Center showed that 72% of Internet users in the United States searched for health information the previous year, with 35% admitting to using the Internet to try to determine their own or someone else’s medical condition [2].

More specifically, Makovsky’s *Pulse of Online Health* in 2015 found that 66% of the US population reported interest in using mobile apps to manage their health [3]. Accordingly, in 2017 over 325,000 mobile health-related apps were commercially available for download, a 25% increase from 2016 [4]. Unfortunately, reviews of these commercially available health apps frequently lack an evidence base or adherence to guidelines, with very few going through clinical trials [5,6]. Moreover, clinical research has struggled to study and define clinical guidelines for the new data collected [7-9], even with exciting opportunities to engage hard-to-reach populations and provide innovative care [10]. Over 50% of individuals in developed countries have at least one chronic disease [11], and about one-fourth of them experience limitations in their activities of daily living [12].

One such chronic disease, inflammatory bowel disease (IBD), which is composed of Crohn’s disease (CD) and ulcerative colitis (UC), is well-situated for technological intervention. By engaging, educating, and monitoring patients, technology can help us understand and improve care in a disease that presents uniquely in each individual. IBD has a 0.5% prevalence for both CD and UC in the western world [13], a number expected to increase globally, with specific spikes in certain populations [14]. IBD is relapsing and remitting in nature, with disease exacerbations (ie, flares) being a key driver of the acute need for medical care [15] and having a negative impact on quality of life (QoL) [16]. Treatment is unique to each patient’s circumstance, including self-monitoring and behavioral interventions [17,18]. Some long-term intestinal complications of CD include strictures, fistulas, and abscesses [19,20]; some long-term intestinal complications of UC are perforation, colitis, colonic strictures, and colorectal cancer [20-23].

The Selecting Therapeutic Targets in Inflammatory Bowel Disease (STRIDE) recommendations and the CALM trial have sparked significant changes in clinical management, suggesting that resolution of symptoms alone is not a sufficient outcome; objective evidence, such as fecal calprotectin (FC) or C-reactive protein, is also necessary to guide clinical decisions [24,25]. Using the objective marker, FC, and patient symptoms to guide therapeutic decisions, 46% of patients in the CALM trial reached mucosal healing after 48 weeks compared to 30% of control subjects [25]. As a result, the goals of treatment are evolving from symptom-based to inflammation-based (ie, symptom and biomarker) control to slow or even reverse disease progression. Due to this shift, patients and providers are challenged to find new ways to engage in monitoring, set goals for treatment, and discuss longitudinal care. Digital health can help close these gaps.

Prior reviews have discussed both clinically tested and commercially available digital health interventions in IBD by listing out apps or discussing the various kinds of technological interventions [26-29]. This review includes some new digital health apps and will focus on how they have fit into clinical care. By highlighting areas where they have been tested and shown promise in the clinical flow, this review builds the foundation for clinicians and technologists contemplating new apps in IBD.

## Methods

### Scoping Review

Scoping reviews embrace systematic discovery and selection of literature to elucidate and summarize the depth and breadth of a field of interest. One of the benefits of creating this review is to allow commentary regarding potential gaps or areas for innovation. Given this background, we believed a scoping review was most appropriate, using the most current guidelines to (1) identify the research question, (2) identify relevant studies, (3) select relevant studies, (4) chart the data, and (5) collate and summarize the results [30,31].

### Development of Research Questions

The research questions were as follows: What digital health apps have been described in the published literature in the setting of IBD? How have these apps complemented or been used in current clinical practice?

### Identifying Apps Discussed in Published Literature

The literature was reviewed using PubMed, Scopus, and ClinicalTrials.gov for articles or trials published from January 1, 2010, through April 19, 2019; published articles and trials were accessed on April 19, 2019. The search terms were “mobile health” OR “eHealth” OR “digital health” OR “smart phone” OR “mobile app” OR “mobile applications” OR “mHealth” OR “smartphones” AND “IBD” OR “Inflammatory bowel disease” OR “Crohn's Disease” OR “Ulcerative Colitis” OR “UC” OR “CD”. Only English-language articles were included. The Scopus search included titles, abstracts, and keywords. The PubMed search included the entire paper and excluded “UC” and “CD” in the search terms. The ClinicalTrials.gov search included studies containing these terms. All papers were reviewed by the first author of this paper (ALY), with consultation from other authors when necessary.

### Identifying Commercially Available Apps

The iTunes iOS app store was reviewed to find a selection of commercial apps for the purpose of discussion and recognition of the large consumer market that patients face. As the review of commercial apps was not intended to be exhaustive, Google Play and other app stores were not explored. The search terms “inflammatory bowel disease,” “crohn’s disease,” “ulcerative colitis,” and “colitis” were used to identify potential apps; the iTunes iOS app store was last accessed on April 30, 2019. Considering the desired length and scope of this review, a restricted number of apps were included. These were selected based on top search *hits* in the app store, which reflect commercial apps that have historically been used most [29].

### Article Selection

The titles and abstracts of all *hits* were reviewed. Articles were included if they explored the use of a digital health intervention in the care of IBD, or CD or UC specifically. Citations of included articles were then also screened for additional relevant articles or apps. Articles were excluded if they did not involve IBD, CD, or UC. They were excluded if they discussed teleconferencing or video chatting as the sole intervention, as this was decided to be out of the scope of the review.

### Data Charting

The articles were organized according to the apps that they described. Each app was assessed for how it addressed any of five identified areas of clinical care: education, monitoring, treatment, follow-up, and patient satisfaction.

### Collation and Summary

We summarize and present the relevant features of the apps by breaking them into the five areas of clinical care identified, providing a framework for each of these areas. The goal of the scoping review was to summarize the depth and breadth of digital health apps that have been used in IBD clinical care in order to provide a foundation for discussion for physicians and technologists to pursue future opportunities.

## Results

### Overview

**Figure 1:**
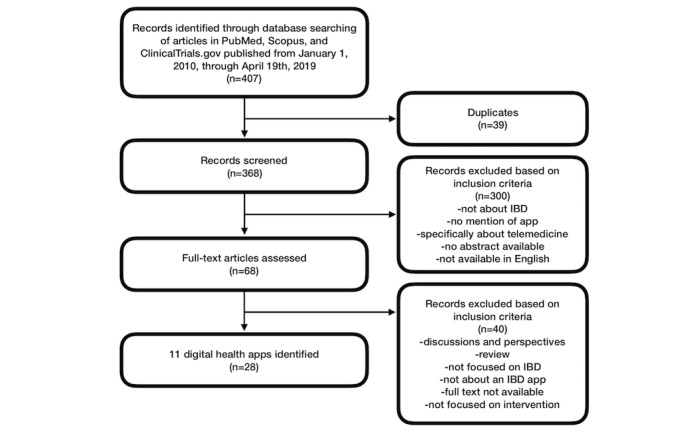
Selection process for articles about inflammatory bowel disease (IBD) digital health apps.

The Scopus search yielded 227 *hits*. The PubMed search returned 168 *hits*. Search of ClinicalTrials.gov returned 12 *hits*. This totaled 407 *hits* from the three databases. A total of 39 duplicates were identified. The titles and abstracts of the remaining 368 articles were reviewed; 68 were identified to meet the inclusion criteria and were examined fully, after which 28 articles were selected (see Figure 1). Exploring citations revealed further information to be included. In total, 11 digital health apps and 4 commercial apps were included.

The five areas of clinical care discussed are education, monitoring, treatment, follow-up, and patient satisfaction. The sections below provide an overview of each area, a review of the clinically studied apps, and a review of a small selection of commercially available apps. An overview of each clinically studied app and the trials’ significant findings can be found in Table 1 [26,32-55]. An overview of each commercial app can be found in Textbox 1 [56-59]. An overview of the features of each app as related to clinical care can be found in Tables 2 and 3.

**Table 1:** Overview of clinically studied apps.

App (sample size)	Feature highlights	Significant findings from trials
Constant Care (n=333, n=95, and n=17) [32-35]	Weekly disease activity: SCCAI^a^ and FC^b^ results are mapped to “traffic light” color score Educational modules Portal for providers	Fewer outpatient and acute care visits Shorter flares and improved QoL^c^ and medication adherence Timely administration of medications Individualized infliximab timing without compromising QoL
Young Constant Care (n=50) [36,55]	Same as Constant Care, with one change: disease activity measured with Pediatric Crohn’s Disease Activity Index (PCDAI) or Pediatric Ulcerative Colitis Activity Index (PUCAI)	Individualized infliximab timing without compromise of QoL Fewer doses given and longer average treatment interval
HealthPROMISE (n=320) [37-41]	Biweekly disease activity (SIBDQ^d^) and QoL (EQ-5D^e^), graphically represented over time Track health items such as vaccines, screenings, etc, to assess overall QoC^f^ Integrated into medical records Patients can see the current plan and message their care team	Improved QoL and QoC over 575 days Fatigue and tension drivers of QoL Patients report more equitable decision making 75% still use at 6-month follow-up
IBD-live (n=84) [42,43]	*Flarometer* portal collects PUCAI or PCDAI and FC at 1- or 3-month intervals Communication module directly connected to local IBD^g^ health team Questionnaire module with surveys about QoL, absenteeism, and health care use Patients and specialists alerted to out-of-range results	No difference in flare occurrence Improvement in QoL, although not reaching significance Fewer face-to-face interactions with providers
myIBDcoach (n=909) [44,45]	Weekly or monthly monitoring modules based on disease severity *Monitor* *IBD at Home* questionnaire to assess disease activity eLearning (electronic learning) module accompanies every session Meeting disease activity thresholds will auto-alert health care providers Patients can see their care plan and message their care team	No change in QoL, flares, acute care visits, and QoC Higher treatment adherence Fewer outpatient visits and telephone calls to physicians Fewer hospitalizations 94% still using at 1-year follow-up
Telemonitoring of Crohn’s Disease and Ulcerative Colitis (TECCU) (n=63) [46,47]	Three-armed randomized controlled trial: control, nurse-assisted telephone care, and app group App group: additional education, reminders, and prompted by text to report symptoms App group: based on reported symptoms, developed alerts and treatment guidelines delivered to patients through the platform	Disease activity and remission status improved most in app-monitored group QoL and medical adherence improved in all groups Differences in work productivity and activity impairment did not reach statistical significance Fewer calls and outpatient visits in app group Improved satisfaction in app and control groups
TELE-IBD (n=348 and n=219) [48-50]	Text message about symptoms (HBI^h^ and SCCAI) and medication side effects Remote changes in management possible if alert thresholds of disease are met Texts to share information about medications, dosing, and frequency	Improved disease activity and QoL in controls and users Decrease in hospitalizations, but increase in electronic encounters, phone calls, and noninvasive diagnostic tests No significant change in knowledge as measured by the CCKNOW^i^ after adjusting for confounding variables
TrueColours UC (n=66) [51]	Daily monitoring (SCCAI) sent by email, biweekly QoL (EQ-5D), and monthly FC “Traffic light” monitoring and presentation of disease state Able to input blood, pathology, endoscopy, and histology results Treatment guidance given regarding 5-ASA^j^ and topical rectal medications	Algorithm predicts escalation in therapy with 95% accuracy Patients reported feeling empowerment, improved awareness, and communication
UC HAT/HAT (n=25 and n=47) [52,53]	Weekly reporting of symptoms, well-being, medications, side effects, and weight Web-based clinician portal Users are able to message their team and print an action plan	No difference in QoL, disease activity, or medical adherence 56% (14/25) completion in UC HAT^k^ 91% adherence to HAT^l^ over 6 months and 86% report no interference with daily routines
UCLA eIBD (n=194 UC^m^ and n=217 CD^n^) [26,54]	Only available to UCLA^o^ IBD patients Disease activity monitored with UCLA-developed four-question PROs^p^ (mHI^q^) with alarms built in at set thresholds Integrates PROs into medical records Value quotient measures patient value vs cost over time	Correlation of mHI with HBI and partial Mayo score for CD and UC, respectively Inverse correlation with QoL as measured by SIBDQ

^a^SCCAI: Simple Clinical Colitis Activity Index.

^b^FC: fecal calprotectin.

^c^QoL: quality of life.

^d^SIBDQ: Short Inflammatory Bowel Disease Questionnaire.

^e^EQ-5D: EuroQol-5 Dimension questionnaire.

^f^QoC: quality of care.

^g^IBD: inflammatory bowel disease.

^h^HBI: Harvey Bradshaw Index.

^i^CCKNOW: Crohn’s and Colitis Knowledge Score.

^J^5-ASA: 5-aminosalicylate.

^k^UC HAT: home automated telemanagement in ulcerative colitis.

^l^HAT: home automated telemanagement.

^m^CD: Crohn’s disease.

^n^UC: ulcerative colitis.

^o^UCLA: University of California, Los Angeles.

^p^PRO: patient-reported outcome.

^q^mHI: Mobile Health Index.

**An overview of commercial apps and their key features.**

GI Monitor [56]

- Log symptoms, meals, bowel habits, medications, and mood
- Tries to help users make insights into behavior and symptoms
- Data can be exported to be shared with physicians

ibd.care [57]

- Website with basics of inflammatory bowel disease (IBD), tips for talking with a care team, advice about lifestyle, and insurance tips
- Parallel site for physicians to learn about insurance and IBD management
- Created by PRIME, a recognized provider of continuing medical education, reviewed by the Academy of Managed Care Pharmacy, and supported by the Case Management Society of America

myIBD [58]

- Mobile app built to assist pediatric patient transition to adult care
- Learn module with videos, articles, and short quizzes
- Journey module for input of features related to disease history: diagnosis, physicians, medications, allergies, major events, goals, symptoms, etc
- Access to disease activity questionnaires that can be saved
- Roadmap of the illness can be shared with new physicians

Oshi [59]

- Shares wellness and symptom scores and insights related to associations with flares
- Tracks symptoms and behavioral information (eg, exercise) and can sync to fitness devices
- *Learn* component has articles, IBD-friendly recipes, and personal stories
- *Ask* component provides a space to message Oshi health professionals

**Table 2:** An overview of the features of each clinically studied app as related to clinical care.

App (sample size)	Clinical care features				
	Educate	Monitor	Treatment	Follow-up	Patient sentiments
Constant Care (n=333, n=95, and n=17) [32-35]	Disease-specific lecture at onboarding eLearning (electronic learning) modules eHealth nurse Video clips	SCCAI^a^, HBI^b^, and FC^c^ “Traffic light” system	Can suggest treatment for flares and maintenance Portal for providers to track patients	N/A^d^	First trial (n=333): 88.8% said system was feasible and preferred using it over standard clinical care Second trial (n=95): 100% were satisfied Third trial (n=17): high satisfaction
Young Constant Care (n=50) [36,55]	eLearning modules eHealth nurse Video clips	PUCAI^e^ and PCDAI^f^ Height and weight “Traffic light” system	Timing of infliximab maintenance therapy Portal for providers to track patients	N/A	74% of surveys completed over the course of the trial
HealthPROMISE (n=320) [37-41]	N/A	SIBDQ^g^ and EQ-5D^h^ every 2 weeks QoC^i^ (vaccinations, and routine check-ups) Integrated into EMR^j^	N/A	Can message care team through platform App displays plan of care	Maintained 75% use at 1-year follow-up
IBD-live (n=84) [42,43]	N/A	*Flarometer* (PUCAI or PCDAI + FC) every 1 or 3 months Disease state shown to patient and team	N/A	Platform indicates when to see care team Can message local IBD^k^ team through platform	96% reported time-savings 71% wished to continue Highly compliant patients averaged €360 in annual savings
myIBDcoach (n=909) [44,45]	Interactive eLearning modules	Monthly modules about disease, medication, satisfaction, and side effects Uses MIAH^l^ survey	At symptom threshold, red flag nudges health care worker to check in	Can communicate with their health care team Can view personal health plan	In largest trial (n=909), 94% continued to use at 1-year follow-up No significant difference in satisfaction vs controls
Telemonitoring of Crohn’s Disease and Ulcerative Colitis (TECCU) (n=63) [46,47]	Educational material created by the researchers received through the platform	Symptoms assessed through texts and questionnaires Disease activity (HBI for CD^m^ and SCCAI for UC^n^)	Reminders sent through platform Automatic alerts to care managers	Could send messages through the platform to health team	Significant increase in satisfaction (modified Client Satisfaction Questionnaire) No perceived breaches in privacy
TELE-IBD (n=348 and n=219) [48-50]	Educational tips delivered through text message, both about general health and IBD specifically	N/A	Remote changes in management if symptom burden is met	N/A	13.9% did not complete every other week 19% did not complete every week
TrueColours UC (n=66) [51]	N/A	SCCAI daily EQ-5D every 2 weeks FC monthly	Guidance about 5-ASA^o^ dose and topical rectal medications Index predicting need for escalation of therapy	N/A	N/A
UC HAT/HAT (n=25 and n=47) [52,53]	Weekly educational packages	Weekly symptom diary, medication effects, and weight	Customized action plan based on reported symptoms	Option to message their health care team	56% (14/25) completed UC HAT^p^ 91% continued use of HAT^q^ over 6 months and 86% report no interference with daily routines
UCLA eIBD (n=194 UC, 217 CD) [26,54]	N/A	Self-developed four-question PRO^r^ questionnaires	Automated message to coordinator at certain disease threshold	Contact a coach through the app	N/A

^a^SCCAI: Simple Clinical Colitis Activity Index.

^b^HBI: Harvey Bradshaw Index.

^c^FC: fecal calprotectin.

^d^N/A: not applicable.

^e^PUCAI: Pediatric Ulcerative Colitis Activity Index.

^f^PCDAI: Pediatric Crohn’s Disease Activity Index.

^g^SIBDQ: Short Inflammatory Bowel Disease Questionnaire.

^h^EQ-5D: EuroQol-5 Dimension questionnaire.

^i^QoC: quality of care.

^j^EMR: electronic medical record.

^k^IBD: inflammatory bowel disease.

^l^MIAH: monitor IBD at home.

^m^CD: Crohn’s disease.

^n^UC: ulcerative colitis.

^o^5-ASA: 5-aminosalicylate.

^p^UC HAT: home automated telemanagement in ulcerative colitis.

^q^HAT: home automated telemanagement.

^r^PRO: patient-reported outcome.

**Table 3:** An overview of the features of each commercial app as related to clinical care.

App	Clinical care features		
	Educate	Monitor	Follow-up
GI Monitor [56]	N/A^a^	Symptom diary: bowel movements, stress, meals, weight, pain, and medications	Can export data to share with physician
ibd.care [57]	Modules: overview of IBD^b^, treatment options, choosing therapy, managing IBD, navigating insurance, and education for providers	N/A	N/A
myIBD [58]	Teaching videos Topic-focused articles Self-assessments	Can download and complete SIBDQ^c^, SCCAI^d^, HBI^e^, and PHQ-9^f^	Can share records in app with provider
Oshi [59]	Educational articles written by MDs (ie, physicians), PhDs, and patients	Symptom diary: bowel movements, abdominal pain, overall well-being, stress, diet, sleep, and exercise	Offers to contact physician if symptoms change significantly *Ask* component allows messaging of Oshi health professionals

^a^N/A: not applicable.

^b^IBD: inflammatory bowel disease.

^c^SIBDQ: Short Inflammatory Bowel Disease Questionnaire.

^d^SCCAI: Simple Clinical Colitis Activity Index.

^e^HBI: Harvey Bradshaw Index.

^f^PHQ-9: 9-item Patient Health Questionnaire.

### Education

After initial diagnosis, patients may learn more about their disease through interactions with their health team, materials provided by clinical providers, discussions with family or friends, or support groups, but the Internet is also a common source of information. Some physicians encourage patients to use curated websites as sources of information. Patient education benefits QoL and continuity of care, reduces patient anxiety and complications from illness, and increases treatment adherence [60]. After diagnosis, up to 86% of individuals diagnosed with a chronic condition will turn to the Internet for information [61], but it is generally accepted that IBD information on the Internet may be too hard to read and have inaccuracies [62-65].

The app myIBDcoach utilizes interactive eLearning (electronic learning) modules about medications, adherence, smoking cessation, nutrition, symptom management, fatigue, work productivity, anxiety, and depression [44]. In addition to eLearning modules, Constant Care gives a disease-specific lecture during the onboarding process and provides access to an eHealth nurse and educational video clips. Constant Care researchers report that this has been valuable in empowering IBD patients to perform individualized, self-administered therapy [35]. At the end of 12 months, a study of 333 participants showed significant improvement in general knowledge about IBD (*P*<.001) and medications (*P*=.001) in the Danish cohort of the study compared to controls, as measured by the Crohn’s and Colitis Knowledge Score (CCKNOW); however, the same effects were not observed in the Irish cohort [32,66]. Home automated telemanagement in ulcerative colitis (UC HAT) developed its own curriculum based on materials from the Crohn’s and Colitis Foundation of America (CCFA), providing educational packages with each weekly check-in [52]. Telemonitoring of Crohn’s Disease and Ulcerative Colitis (TECCU) produced its own educational and preventative materials that were incorporated and available on the platform [46].

TELE-IBD used a text message-based curriculum to send educational information at various frequencies, providing tips related to both general (ie, vaccinations and screenings) and specific (ie, IBD medication side effects) health information. In a study of 219 participants, patients were randomized to receive either TELE-IBD messages at different frequencies or standard of care. Results measured by CCKNOW showed significant improvement in patients receiving messages every other week as compared to controls (*P*=.03) and greater changes in scores in participants with lower baselines (*P*<.01); however, after adjusting for race, site, and baseline, researchers found no significant changes between control and TELE-IBD groups [48-50].

Of the commercial apps, myIBD, though mainly built for transition of care between providers, contains a robust educational component with videos, short articles on many related topics, and quizzes to assess knowledge [58]. The app ibd.care is created by PRIME, a recognized provider of continuing medical education, reviewed by the Academy of Managed Care Pharmacy, and supported by the Case Management Society of America. This app covers a broad base of information about IBD, including basics of disease, treatment, tips for talking with your care team, navigating insurance, and how to align lifestyle with IBD goals. The site similarly aims to educate health care providers about managing patients and navigating insurance [57]. Many apps direct patients to the CCFA, where a large breadth of curated IBD information is available [20].

The apps approach education through learning modules, videos, and automated text messages covering a wide array of IBD topics. Besides the TELE-IBD study, these apps have acted as a repository for educational materials to be accessed by patients when needed or when the platforms are used, but the materials are not delivered proactively.

Although education is a common component across almost all apps, there is minimal assessment of the information quality or the value that the information provides to patients. It would be interesting to assess whether patients using these apps rely less on unverified sources of information and, as a result, experience improvements in treatment adherence, QoL, anxiety, and complications. With the varieties of technology available, technologists may be able to engage with physicians to create more interactive and engaging educational materials that increase health literacy and empower patients in managing their care.

### Monitoring

Generally speaking, monitoring a patient’s symptoms occurs at discrete time points at certain frequencies (ie, they can vary from every few months to every year) through outpatient office visits, when physicians and patients discuss symptoms experienced since a prior visit. The subjective reports from patients and laboratory data may both be collected at these time points to monitor the state of a patient’s disease. Understanding the symptoms that patients experience is a foundational component of clinical care, but physicians are not always effective at collecting this information [67]. One study observed that health care providers frequently misinterpret reported symptoms, leading to differences in how the patient and the physician perceive a patient’s current state [68]. As a further challenge, the concordance between a patient’s memory of experience and actual experience of gastrointestinal (GI) symptoms has been observed to be generally poor [69]. The insufficient identification and management of IBD flares is one reason that many patients have poor outcomes [49].

Using a Web-based monitoring package, Constant Care maps symptoms reported through the Simple Clinical Colitis Activity Index (SCCAI) and Harvey Bradshaw Index (HBI) questionnaires, along with fecal calprotectin (FC) measurements, to a “traffic light” color system, which illustrates disease activity based on a total inflammatory burden score. Patients with recent flares take the surveys daily or weekly until they enter the *green zone* (ie, remission). Patients already in the *green zone* report symptoms monthly. Studies involving Constant Care have shown improved QoL, shorter flares, and decreased acute care and office visits [26,28,32].

HealthPROMISE tracks patient symptoms and QoL biweekly using the Short Inflammatory Bowel Disease Questionnaire (SIBDQ) and the EuroQol-5 Dimension questionnaire (EQ-5D), respectively. Providers can view the data on a Web-based dashboard integrated with this provider’s electronic medical record (EMR). Due to the integration, researchers believe that in-person office visits can focus more on quality of care (QoC) as opposed to eliciting symptom history, allowing for more meaningful goal-focused discussions [37]. In their trial with 320 participants, the results showed *fatigue* and *tension* as the most important drivers of QoL [41], with QoL having significantly improved for study patients as compared to controls after 575 days of follow-up [39].

IBD-live is a Web-based app comprised of three modules that were tested on adolescents. The first module focuses on monitoring using a *flarometer*, where patients report disease activity through the Pediatric Ulcerative Colitis Activity Index (PUCAI) or the Pediatric Crohn’s Disease Activity Index (PCDAI) and send an FC sample to track their status, as mapped to a “traffic light” system. Patients receive email reminders to report into the module. In the study, the 84 users of the platform had no difference in experiencing flares (33% vs 34% in controls) but showed some improvement in QoL (+1.32 vs -0.32 in controls, measured using the IMPACT-III questionnaire, *P*=.27) [42,43].

In TECCU, the 21 people in the intervention group were monitored via an IBD-modified version of NOMHADhome, a technological system designed for managing chronically ill patients and accessible on computers, tablets, or mobile phones. Patients reported symptoms by answering questions sent via text message and accessed questionnaires on the platform to monitor disease activity, adverse effects, and medication adherence. Monitoring frequency varied depending on the therapy plan for each patient, ranging from every week to every 4 weeks. Measurement of disease activity (HBI for CD or SCCAI for UC), QoL (Inflammatory Bowel Disease Questionnaire 9), and productivity and activity impairment (Work Productivity and Activity Impairment questionnaire) were done at the beginning and the end of the 24-week study. Researchers observed greater improvement in disease activity in the TECCU group compared to standard care, but the results did not reach statistical significance. QoL and social impairment improved significantly in all groups in the study, with no significant differences between them [46,47].

In a study with 47 participants, UC HAT used a symptom diary to elicit information on weekly symptoms, medications, side effects, and weight. The study did not find significant improvement in disease activity or QoL over standard care [52]. The app myIBDcoach has a monthly monitoring module to collect information about disease activity as measured with the Monitor IBD at Home questionnaire, medication use, treatment adherence, treatment satisfaction, and side effects. In a study with 909 participants using myIBDcoach, there was no significant difference in disease activity [44]. UCLA eIBD developed two specific, four-question, patient-reported outcome (PRO) questionnaires, named the Mobile Health Index (mHI), that are integrated into the EMR and used to create a patient value quotient. A study of 194 UC and 217 CD patients compared their mHI to standard UC and CD clinical disease activity measures, showing significant correlation for each (*P*<.001). Both had a strong inverse correlation with QoL as measured by the SIBDQ (*P*<.001) [29,54]. TrueColours UC emails users to report symptoms every day through the SCCAI and to report on QoL every 2 weeks through the EQ-5D. Combined with a monthly FC test, users tracked disease activity using the system’s “traffic light” severity tracker [51].

In the commercial market, symptom diaries represent about 57% of the IBD-related apps available for English speakers on the Google Play and Apple app stores [29]. Apps like Oshi, GI Monitor, and myIBD have various methods to track symptoms, bowel movements, medications, meals, emotional state (ie, stress and anxiety), sleep or fatigue, and physical activity. In return, these apps graphically represent the data to help users track and potentially discover associations between symptoms and reported information [56,58,59].

All but two of the apps included in this review have some approach to collecting PROs. Most apps going through trials tend to collect data at discrete, weekly, biweekly, or monthly time points, using standard clinical questionnaires for IBD. Conversely, commercial apps tend to aim for daily data entry and ask patients to report more granular data (eg, specific symptoms, activities, and emotions). No studies have reported decreases in QoL as a result of app use, and many—Constant Care, HealthPROMISE, and TECCU—have reported improved QoL.

### Treatment

IBD management, for both UC and CD, includes the following: induction therapy and maintenance therapy. The goal is first to control inflammation quickly and then to sustain that control over time. Symptoms, side effects, laboratory data, and imaging inform the choice of therapy. Clinical treatment guidelines provide algorithms for physicians to follow based on patient symptoms, laboratory data, and prior treatments. The recommended treatments can vary greatly depending on these factors, and the treatments may not consider unique patient treatment preferences or individual characteristics [17,18].

Constant Care can provide recommendations for medication management based on reported symptoms. In one trial with Constant Care, patients with UC who reported acute symptoms indicative of a flare received recommendations to initiate 5-aminosalicylate (5-ASA) for a certain duration. Based on their own prior experience and current treatment guidelines, patients could also select additional treatments. In this trial with 333 participants, 100% of patients in the treatment group received 5-ASA in response to a flare as compared to 10% of control patients. Researchers attributed this benefit to the patient’s ability to better recognize and understand the correct treatment for a flare [32,35]. Constant Care has also been studied in the context of down-titration of mesalazine therapy in mild-to-moderate UC patients in a trial with 95 participants. Patients were encouraged to decrease or maintain their dose or reach out to their care team based on the “traffic light” system. Results showed a significant increase in adherence to mesalazine (Visual Analog Scale 88 vs 100, Medical Adherence Rating Scale 23 vs 24, *P*<.001) in the group from baseline to the end of the study; results also showed significantly improved QoL (*P*<.001) as measured by the SIBDQ and 12-Item Short Form Health Survey (SF-12) from baseline to the end [33]. A third trial with 17 participants used Constant Care to determine the timing of infliximab maintenance therapy for CD, using weekly entries converted to the “traffic light” system. At the end of the trial, only 10% of individuals received therapy at 8-week intervals (ie, standard of care), 39% received treatment at shorter intervals, and 50% received treatment at longer intervals [34].

A similar randomized controlled trial of 50 patients used Young Constant Care—an adapted version of Constant Care—in a pediatric population. Based on symptom reporting and FC, patients reaching the *red* level or 2 weeks of the *yellow* level received an infusion of infliximab (ie, standard maintenance care). Otherwise, patients were allowed a maximum of 12 weeks between infusions. Using these methods, researchers observed a significant increase in the mean treatment interval (9.5 weeks vs 6.9 weeks, *P*<.001) and no change in QoL or levels of antibodies between treatment and controls who received standard care [36].

In TECCU, the responses provided through the platform were used to create individually tiered alerts and action plans. Patients received recommendations to adjust medication or visit their physician based on the alert level matching their reported symptoms. At the end of the trial, the HBI for CD or the SCCAI for UC were combined with partial Mayo scores from face-to-face visits to assess remission status. After 24 weeks, the 21 patients in the intervention group had no significant change in remission in UC (odds ratio [OR] 0.12, *P*=.19) or CD (OR 0.11, *P*=.13) or in mean improvement in measured FC (-0.90 mg/g, *P*=.11) [46,47].

In HealthPROMISE, patients have reported feeling that their care decision making has been more equitable and have experienced improved health outcomes (QoC increased +19% more than controls, *P*<.01) [40]. TrueColours UC used the data collected during its study of 66 participants to create a flare algorithm to help predict the need for escalation in therapy at outpatient visits and observed 95% accuracy of this algorithm [26].

Current evidence about the role of apps in IBD treatment comes largely from four different studies involving the Web-based platform Constant Care. Suggesting the inception or alteration of a medication through the use of IBD digital health apps has been viable. Certain studies have shown significantly increased QoL and have explored individualizing treatment timing. None of these studies have reported worsening QoL, QoC, flares, or treatment outcomes in participants.

The uses of Constant Care to titrate treatment for flares or maintenance therapy are exciting and have been recognized as an early step in the pursuit of pharmacokinetic monitoring [70]. One single treatment of a biologic therapy like infliximab can cost thousands of dollars and require an entire day in an infusion center [71]. The prospect of individualizing treatments according to when they are needed could benefit patients (ie, reduced medication exposure and time lost from daily life) as well as the health care system (ie, cost avoidance, fewer visits, and fewer side effects). Further studies are needed to validate this potential in IBD digital health apps.

### Follow-Up

After diagnosis, patients with chronic diseases will regularly visit their physician to share updates about symptoms and side effects. Unexpected or worsening symptoms may warrant scheduling additional visits to alter or add therapies. Severe changes may require visits to the emergency room or hospitalizations for immediate care. Higher numbers of outpatient visits can serve as a significant protective factor against IBD-related hospitalization in the following year [72], but nonadherence to medication is common in IBD and reported to be in the range of 40%-60%, with related adverse economic and clinical implications [73]. Appropriate continuity of medical care is associated with higher patient satisfaction, fewer hospitalizations, fewer emergency room visits, and improvement in receiving preventative services [74].

During testing of the Constant Care app, researchers observed an increase in the amount of contacts over the phone and through email, but a reduction in visits to the emergency department and the same number of hospitalizations [28]. The Constant Care trial also noted improved adherence to medication during flares (73% vs 42%, *P*=.005) [32]. UC HAT had a messaging option to a nurse coordinator through their Web portal but showed no changes in adherence and did not assess visits or hospitalizations [52]. Over 12 months, the trial of the myIBDcoach system showed a significant reduction in both visits to the gastroenterologist (1.55 vs 2.34 average visits over a year, *P*<.001) and telephone calls to the gastroenterologist (0.58 vs 0.84 average calls over a year, *P*<.001) compared to controls; however, there was no change in visits (0.29 vs 0.36, *P*=.17) or telephone calls (0.70 vs 0.74, *P*=.45) to the nurse. Users of the system had fewer hospitalizations (*P*=.046) and higher medication adherence, as measured by the Morisky Medication Adherence Scale (*P*<.001) [44].

In TECCU, patients could use the platform to send messages to their teams and receive direction about when to follow up or adjust medications. Researchers observed a significant increase in medication adherence in all groups in the trial, with significantly higher increase in the patients in the app group as measured by the Morisky-Green index (OR 0.0001). Researchers noted lower numbers of outpatient visits and telephone calls in the intervention group compared to controls [46,47].

Users of the IBD-live platform were triaged to different follow-up plans depending on their reported symptoms. Patients considered low risk would report symptoms again in 3 months, those at intermediate risk would report again in 1 month, and those at high risk would be directed to contact their physician. Patients were also able to contact their local health team through the platform. Among the 84 users of the Web-based system, there were significantly fewer face-to-face follow-ups as compared to controls (3.6 visits vs 4.3 in controls, *P*<.001) [42,43].

Among the commercial apps, MyIBD, aims to support the transition from a pediatric to an adult gastroenterologist. Problems with transition from pediatric to adult care can lead to treatment nonadherence, increased disease severity, and undue emotional and financial stress for patients [75]. Data show that 79% of adult gastroenterologists report inadequate preparation of adolescents coming from pediatric care [76]. The MyIBD app provides a *My Journey* module where patients can record all aspects of their own health record and grant access to new physicians they meet in their care [58]. Oshi has a feature where users can give the app permission to contact their physician if reported symptoms may indicate worsening disease [59].

The above apps provide another way for patients to interact with their care, whether through direct messaging or the ability to view information about their disease. Medication adherence is generally improved in the studied app users. These users also benefit from decreases in acute care and outpatient visits, although results are mixed about changes in telephone contact with providers. No studies reported increased hospitalizations or emergency visits or decreased medication adherence.

### Patient Satisfaction

There is no globally agreed-upon formulation for patient satisfaction [77], and determinants of patient satisfaction vary across different studies, providing little explanation for the influencers of satisfaction [78-80]. One review of mobile health in managing digestive diseases determined that patient satisfaction ranged from 74% to 100% in the reviewed studies, with compliance ranging from 25.7% to 100% [81]. IBD patients want to be involved in decision making, with many reporting the desire for equitable collaboration with their physician [82]. In one set of focus groups, IBD patients reported a lack of understanding of how well their disease was being controlled, including a feeling that QoL was not discussed in many visits with their physicians. The patients reported an overuse of jargon and felt a lack of tangible goals or goal-setting in their care [83].

At the end of the largest Constant Care trial, 88.8% of individuals said the system was feasible and wanted to continue using it [32]. In another trial, 100% of individuals who finished the study reported being satisfied [33]. In a third trial, users reported high satisfaction with Web programs, education, and impact of the program on their disease [34]. The app myIBDcoach noted that 94% of users continued using the platform at the 1-year follow-up but saw no significant difference in satisfaction, as measured by a Visual Analog Scale in Web platform patients versus those receiving standard of care [44]. For UC HAT, only 14 of the 25 (56%) participants completed the study [52]; in the following version, home automated telemanagement (HAT), 86% reported that using the system did not interfere with their daily routines, 91% would consider using it in the future, and 91% were adherent to using the platform for 6 months [53]. In TELE-IBD, a posttrial qualitative study of both adherent and nonadherent patients identified benefits of understanding disease, monitoring symptoms, and feeling connected to their health provider. This study also noted that many participants had trouble remembering details of their action plans and that there were mixed results regarding timing, repetition, and technical aspects of the platform [84]. HealthPROMISE continued to have 75% adherence after 6 months of follow-up [37,41]. Participants of the Young Constant Care trial completed 74% of total desired survey entries [55]. In TECCU, patients in the control and intervention groups reported significant increases in satisfaction from baseline, as measured by an adapted version of the Client Satisfaction Questionnaire. Patients and providers also reported no perceived privacy breaches and minimal technical issues [46,47]. In IBD-live, 96% of users reported the platform to be time-saving, 71% wished to continue, and highly compliant patients averaged €360 in annual savings [43].

Patients must remain the center of iteration and development. App adherence in the above studies usually means that users continue to use the app over the time frame stated, but researchers have not described the quantity of use and its possible relationship with patient progress. Although adherence has generally been adequate and satisfaction generally positive, studies have described little beyond these simple measures: a shortcoming observed across much of digital health [85]. As in the TELE-IBD posttrial study, narrative feedback will be important in improving specifics related to apps (ie, design and function) and answering larger questions about the perceived shortcomings of interventions. Clinical guidelines are able to advise specific treatments and medications but struggle to account for specific patient characteristics, such as treatment preference, access to care, childbearing interest, age, treatment history, etc. This can lead to disagreements or misunderstandings between patients and physicians. Digital health may be able to empower patients to better understand some of this context and help providers be more aware of patient preferences.

## Discussion

### Principal Findings

As in many chronic diseases, multiple influences—genetics, medications, behavior, social network, environment, psychological factors, and social determinants—play a role in the course of IBD, greatly increasing the number of variables and potential interventions available for study [8,86-88]. In such a large dimensional context, a randomized controlled trial (RCT) could assess whether an intervention, on average, has some effect, but it is unlikely to determine which of the components led to the observed effect. As seen in this review, adherence, QoL, QoC, and knowledge, among others, were valuable outcomes measured in the studies, but determining which components of each app contributed to these measurements poses a difficult challenge.

Given the breadth of variables and opportunities possible with digital health, RCTs have some limits. Microrandomized trials may be an interesting method to explore moving forward, as they randomly assign intervention options at relevant decision points. This allows for assessment of the effects of each intervention, including when and for whom it causes effects, as well as the examination of factors influencing these effects [89]. This may be a path to better understanding effects of individual components of increasingly complicated digital health apps.

New technologies have the potential to change the care of IBD. One such example is augGI, a technology company whose aim is to improve the management of chronic gut disorders. The company augGI is developing technology that uses computer vision and deep learning to characterize stool specimens from just an image. In particular, they are focusing on measuring stool consistency to better characterize motility changes [90]. Another area is the iteration on symptom monitoring. Many of the reviewed apps use written surveys converted into mobile text versions. Making these questionnaires visual, adaptive, or more specific to individuals could make the data more valuable and individually meaningful, as have been used in other areas [91-93]. On the horizon, toilets may be capable of collecting various data on urine and stool, making some data available more frequently [94-96]. Finally, apps for patients to assess FC at home (ie, IBDoc or Calpro) have been developed and have been positively validated, in general [28,97-99].

As promising as these technologies are, they bring to the forefront the lack of clinical guidelines for what types of new data should be collected and the appropriate frequency of data collection. In the CALM trial, FC was measured at 12-week intervals, already an increase in frequency from the norm. But what if FC is regularly measured every week or every day? As these questions arise, it is vital to utilize datasets that may already exist [100] and find new sets that lead to meaningful, cost-effective guidance for patients. Technologists will be challenged to design devices that elicit and present data streams with clinical relevance. Researchers will be challenged to build clinical guidelines and frameworks for translating these data streams into patient recommendations in real time as the data become available. One prospective study is exploring this dilemma using Fitbits to passively collect daily steps, heart rate, and sleep data and to determine if this data can help predict elevation in biomarkers. One of their early findings has shown that decreased physical activity, as measured by steps the week before, has occurred prior to the finding of active disease (*P*<.001) [101].

The clinical care of IBD is shifting from symptom-based to inflammation-based management. As digital health evolves, it becomes hard to ignore its potential to contribute to this shift. Digital health can help engage patients to track both the visible (ie, symptoms they experience) and invisible (ie, FC or other biomarkers) markers of disease. What a patient feels symptomatically is not fully descriptive of their disease state. Clinicians and technologists alike must pursue other lab or digitally trackable biomarkers to better describe the disease state. In tracking these, patients can receive treatment early in the course of flares, thereby reducing the need for acute care and the risk of long-term complications. Longitudinal assessment of FC or other markers will be able to not only reshape treatment but also allow for a more interactive, goal-focused dialogue between patients and providers.

### Summary

This review discussed the role of studied IBD digital health apps and a small sample of commercial apps in clinical care. Significant benefits have been observed in education, QoL, QoC, treatment adherence, and medication management with the use of some apps. While digital health technologies have shown an ability to fit into, complement, and improve the standard clinical care of patients with IBD, research to further validate these findings from both a clinical and patient perspective is needed. As technologies change, research must expand to define new norms for using the different kinds of data that can be collected and integrated into clinical care. As the clinical management paradigm changes from symptom-based to inflammation-based care, it is an important time for all groups involved—patients, clinicians, technologists, insurers, etc—to discuss and explore new opportunities to use digital health to improve understanding of disease, patient experience, and patient care.

## Abbreviations

5-ASA: 5-aminosalicylate
CCFA: Crohn’s and Colitis Foundation of America
CCKNOW: Crohn’s and Colitis Knowledge Score
CD: Crohn’s disease
EMR: electronic medical record
EQ-5D: EuroQol-5 Dimension questionnaire
FC: fecal calprotectin
GI: gastrointestinal
HAT: home automated telemanagement
HBI: Harvey Bradshaw Index
IBD: inflammatory bowel disease
mHI: Mobile Health Index
MIAH: monitor IBD at home
N/A: not applicable
OR: odds ratio
PCDAI: Pediatric Crohn’s Disease Activity Index
PHQ-9: 9-item Patient Health Questionnaire
PRO: patient-reported outcome
PUCAI: Pediatric Ulcerative Colitis Activity Index
QoC: quality of care
QoL: quality of life
RCT: randomized controlled trial
SCCAI: Simple Clinical Colitis Activity Index
SF-12: 12-Item Short Form Health Survey
SIBDQ: Short Inflammatory Bowel Disease Questionnaire
STRIDE: Selecting Therapeutic Targets in Inflammatory Bowel Disease
TECCU: Telemonitoring of Crohn’s Disease and Ulcerative Colitis
UC: ulcerative colitis
UC HAT: home automated telemanagement in ulcerative colitis
UCLA: University of California, Los Angeles
